# Evaluation of brain nerve function in ICU patients with Delirium by deep learning algorithm-based resting state MRI

**DOI:** 10.1515/biol-2022-0725

**Published:** 2023-10-24

**Authors:** Xiaocheng Huang, Ruilai Jiang, Shushan Peng, Yanbin Wei, Xiaogang Hu, Jian Chen, Weibin Lian

**Affiliations:** Department of Respiratory and Critical Care Medicine, Lishui Second People’s Hospital, Lishui, 323000, Zhejiang, China; Department of Psychiatry, Lishui Second People’s Hospital, Lishui, 323000, Zhejiang, China

**Keywords:** deep learning algorithm, resting state MRI, delirium, brain nerve function

## Abstract

The purpose of this study was to explore the value of resting-state magnetic resonance imaging (MRI) based on the brain extraction tool (BET) algorithm in evaluating the cranial nerve function of patients with delirium in intensive care unit (ICU). A total of 100 patients with delirium in hospital were studied, and 20 healthy volunteers were used as control. All the subjects were examined by MRI, and the images were analyzed by the BET algorithm, and the convolution neural network (CNN) algorithm was introduced for comparison. The application effects of the two algorithms were analyzed, and the differences of brain nerve function between delirium patients and normal people were explored. The results showed that the root mean square error, high frequency error norm, and structural similarity of the BET algorithm were 70.4%, 71.5%, and 0.92, respectively, which were significantly higher than those of the CNN algorithm (*P* < 0.05). Compared with normal people, the ReHo values of pontine, hippocampus (right), cerebellum (left), midbrain, and basal ganglia in delirium patients were significantly higher. ReHo values of frontal gyrus, middle frontal gyrus, left inferior frontal gyrus, parietal lobe, and temporal lobe and anisotropy scores (FA) of cerebellums (left), frontal lobe, temporal lobe (left), corpus callosum, and hippocampus (left) decreased significantly. The average diffusivity (MD) of medial frontal lobe, superior temporal gyrus (right), the first half of cingulate gyrus, bilateral insula, and caudate nucleus (left) increased significantly (*P* < 0.05). MRI based on the deep learning algorithm can effectively improve the image quality, which is valuable in evaluating the brain nerve function of delirium patients. Abnormal brain structure damage and abnormal function can be used to help diagnose delirium.

## 1 Introduction

Delirium is a relatively common and refractory complication, nearly 4/5 patients with tracheal intubation will have delirium, and nearly half of patients without tracheal intubation will also have delirium [1,2]. If the diagnosis and intervention measures can be confirmed in time at the early stage of delirium, the prognosis of patients can be effectively improved. Diffusion tensor imaging (DTI) and resting-state brain functional magnetic resonance imaging (MRI) are new imaging methods for evaluating brain function [3,4]. Fractional anisotropy (FA) is a commonly used index for evaluating medical images in DTI [5]. FA reflects the degree of anisotropy of local structure, that is, tissue atrophy, fiber bundle domination, and membrane structure distribution. The higher the FA value, the greater the degree of tissue structure stretching in a certain direction, which corresponds to the main direction of the total diffusion tensor and reflects the connectivity of fiber bundles in this direction [6]. DTI can provide information about the microstructure of tissues by measuring the diffusion of water molecules in tissues. DTI can measure FA, mean diffusivity coefficient (MD), anisotropy, and scattering tensor. In DTI, FA and MD are often used to reflect the microstructure changes of tissues, which are related to tissue damage and dysfunction [7,8]. The changes of FA and MD are manifested in many diseases, such as neurodegenerative diseases, cerebral infarction, and multiple sclerosis.

Resting MRI image is one of the main imaging methods used to study the structure and function of brain neural networks. In the process of static MRI image processing, there are often some problems related to image reconstruction, such as obtaining static MRI images with high spatial resolution and high temporal resolution in a short scanning time, which bring challenges to static MRI image processing and analysis [9,10]. The traditional static MRI image reconstruction algorithm often needs to iterate the sequence many times, which has a large amount of calculation, and the effect of dealing with image noise, artifacts, and other issues is not ideal [11]. Deep learning model can automatically learn and extract features from a large number of data, has strong data modeling ability, can handle a large number of high-dimensional information, and can effectively improve the reconstruction speed and effect of resting MRI images [12]. In recent years, resting MRI image reconstruction algorithms based on deep learning are gradually emerging. Resting MRI image reconstruction algorithm based on the deep learning algorithm can directly learn the spatial characteristics and semantic information of MRI images and can deal with artifacts and other problems in the reconstruction process [13]. The application value of deep learning algorithms in static MRI image reconstruction mainly lies in improving the speed and quality of image reconstruction [14]. Deep learning algorithms can automatically learn the characteristics of MRI images and generate models through training, thus achieving fast and high-quality static MRI image reconstruction, which provides strong technical support for static MRI image processing and analysis [15].

Evaluation of brain function of patients with delirium in intensive care unit (ICU) by resting MRI imaging based on deep learning algorithms can provide a more accurate and objective evaluation method of delirium in ICU by analyzing the brain function characteristics of a large number of patients with delirium in ICU. The novelty of this study provides a more accurate and effective means for the diagnosis and treatment of delirium in ICU by applying the deep learning algorithm to the evaluation of brain function of patients with delirium in ICU, thereby expanding the application of deep learning algorithm in the field of medical image processing and analysis.

## 2 Methods

### 2.1 Research object

One hundred delirium patients (48 males and 52 females, mean age (51.7 ± 8.46)) hospitalized in the hospital from December 2020 to June 2022 were selected. All patients voluntarily signed an informed consent form. This research has been approved by the Ethics Committee of the Hospital.

Inclusion criteria: Delirium was defined as a score of ≥4 on the intensive care delirium screening checklist (ICDSC) (total score 0–8) by two experienced clinical ICU attending physicians and the right-handed determined by the Chinese handedness assessment criteria. Exclusion criteria: Previous neuropsychiatric disorders, familial genetic diseases, etc.; previous severe liver, kidney, and heart disease; cardiac pacemakers or metallic medical implants in the body; deafness, blindness, and other illnesses that may interfere with delirium assessment. Twenty healthy volunteers were as controls, including 9 males and 11 females, with a mean age of (52.8 ± 8.62), right-handed. The two groups had no obvious difference in basic data (*P >* 0.05). Healthy volunteers met the above inclusion and exclusion criteria.

ICDSC score includes the following eight items: consciousness, improvement of oxygenation index, activity level, nocturnal sleepiness level, circadian rhythm response, speech disorder, perceptual disturbance, and age (over 65 years old). Each item has a score standard of 0–2, and the total score ranges from 0 to 8.


**Informed consent:** Informed consent has been obtained from all individuals included in this study.
**Ethical approval:** The research related to human use has been complied with all the relevant national regulations, institutional policies and in accordance with the tenets of the Helsinki Declaration, and has been approved by the Ethics Committee of Hospital.

### 2.2 Magnetic resonance detection method

A 3.0 T magnetic resonance instrument was applied (Signa HDxt, Shanghai Huantong Nuclear Magnetic Equipment Co., Ltd.). A superconducting MRI scanner and an 8-channel cranial dedicated coil were adopted; during scanning, the patient needed to maintain a quiet and relaxed state in the supine position, lay flat breathing, close both eyes, and patient’s mind go blank. Axial T2 Flair images were first collected to rule out organic brain lesions and significant white matter degeneration, and the parameters were as follows: TR/TE = 8,500/120 ms, slice thickness = 5 mm, and slice interval = 1.5 mm; resting-state functional parameters were comprehensively and systematically analyzed during scanning: TR/TE = 2,000/40 ms, slice thickness = 4 mm, slice interval = 0, number of slices = 33, time point = 210, FOV = 24 cm × 24 cm, NEX = 1, matrix = 64 × 64, flip angle = 90°, and a total of 6,930 images were collected; DTI parameters: TR/TE = 8,500/40 ms, slice thickness = 4 mm, slice interval = 0, slice number = 35, FOV = 24 cm × 24 cm, *b* value = 0, 1,000 s/mm^2^, diffusion sensitivity ladder direction = 25, NEX = 1, matrix = 128 × 128, flip angle = 90°, and two b0 images were collected, 1,820 images were acquired.

Data related to resting-state function were analyzed and processed. The method was to use professional statistical software for analysis. The software used in the experiment was a software analysis system in the research field of key laboratories, mainly including DPARSFA and REST. The software can effectively analyze and sort the data and comprehensively control the scanning and imaging, time-slice correction, head movement correction; the white matter scanning signal was removed during the experiment; the data with translation greater than 1.5 mm and rotation greater than 1.5° were removed; spatial standardization; the scanning signal was screened and filtered using the correlation analysis software to remove the low-frequency signal; coefficient analysis and collation was performed through Kendal’s coefficient of concordance (KCC) software to analyze whether a voxel had consistency with 26 voxels in and around it, the analysis results as the ReHo value of this voxel, and a standardized ReHo map was generated by dividing the KCC value of each voxel by the average KCC value of the whole brain; finally, spatial smoothing and denoising were carried out with a 6 mm smoothing kernel.

### 2.3 Image reconstruction based on the deep learning algorithm

The brain essence is analyzed using the brain extraction tool (BET) algorithm. Draw a circle with the center of the brain as the center and \[\frac{{r}_{0}}{m}]\] as the radius. Mark *N* equal points on the circumference to represent the starting point of the outline. Three forces are defined, namely, tension force \[{u}_{1}]\] , smoothing force \[{u}_{2}]\] , and expansion force \[{u}_{3}]\] . The contour point is pushed to the edge of brain tissue to complete brain tissue segmentation. These three forces have the following relationship: \[{u}_{1}]\] keeps parallel to the tangent line of the brain surface, keeping a certain distance between the points on the brain surface. Perpendicular to the tangent line of the brain surface through a certain curvature, \[{u}_{1}]\] makes the brain contour smoother. \[{u}_{1}]\] is perpendicular to the tangent line of the brain surface, so that the surface of the brain can be deformed outward or inward.

If *O* is the center point of the brain contour, \[{A}_{0}]\] , \[{A}_{1}]\] , \[{A}_{2}]\] are the three adjacent equal points on the skull, *AC* is the midpoint of the line \[{A}_{1}{A}_{2}]\] , vectors \[{S}_{n}]\] , \[{S}_{t}]\] are the decomposition vector of the centerline vector *S*, and the vector \[{S}_{n}]\] is orthogonal to point \[{A}_{t}]\] with \[{A}_{1}{A}_{2}]\] . Perpendicular vector \[{\text{S}}_{n}=S-{\text{S}}_{t}]\] . *R* is the radius, and \[{e}_{{\text{S}}_{n}}]\] is the unit vector of \[{S}_{n}]\] . The change process of three forces pushing vertex \[{A}_{0}]\] is shown in the following equations:

(1) \[{u}_{1}={\text{S}}_{t},]\]

(2) \[{u}_{2}={\text{S}}_{n}\left[1+\frac{\text{tan}\left(Fc\times \frac{1}{r-E}\right)}{2}\right],]\]

(3) \[{u}_{3}={e}_{{\text{S}}_{n}}\left[2\times \frac{{I}_{\text{man}}-{t}_{1}}{{I}_{\text{max}}-{t}_{2}}\right],]\]

(4) \[{t}_{1}={t}_{2}+{b}_{t}({I}_{\text{max}}-{t}_{2}),]\]

where *r* \[\hspace{.25em}=\frac{{l}^{2}}{2| {\text{S}}_{n}| }]\] , *l* represents the average distance between the current point and the adjacent point, \[l=\frac{({A}_{1}{A}_{2}+{A}_{0}{A}_{2})}{2}]\] , \[E=\frac{\frac{1}{{r}_{\text{min}}}+\frac{1}{{r}_{\text{max}}}}{2}]\] , \[F=\frac{6}{\frac{1}{{r}_{\text{min}}}+\frac{1}{{r}_{\text{max}}}}]\] . \[{d}_{1}]\] forward along the line *R* on the image, the minimum gray value \[{I}_{\text{min}}]\] can be obtained, and then \[{d}_{2}]\] forward by the same method, the maximum gray value \[{I}_{\text{max}}]\] can be obtained. \[{t}_{1}]\] means the dynamic image threshold, which is determined by the threshold \[{t}_{2}]\] and weight coefficient \[{b}_{t}]\] for distinguishing image and background.

It is found that the relationship among the three forces is: when point \[{A}_{0}]\] moves to \[{A}_{C}]\] , \[{u}_{1}]\] can make the distance between \[{A}_{0}]\] and adjacent point unchanged. \[{u}_{2}]\] determines the smoothness of the contour curve; if point \[{A}_{0}]\] is convex, under the action of \[{u}_{2}]\] , \[{A}_{0}]\] is subjected to an inward force, while if point \[{A}_{0}]\] is concave, the situation is just the opposite. Curvature of \[{u}_{2}]\] and \[{A}_{0}]\] shows a positive correlation pattern. The gray value of the adjacent area of \[{A}_{0}]\] distribution determines the value of \[{u}_{3}]\] ; if \[{I}_{\text{max}}]\] and \[{t}_{1}]\] are close, \[{u}_{3}]\] will become very small. In this case, the resultant force of the three forces will approach zero infinitely ( \[{u}_{1}]\] , \[{u}_{2}]\] , \[\text{ }{u}_{3}]\] are mutually restricted), which means that the contour evolution is over, and the brain parenchyma image segmentation is completed.

It is necessary to register the brain images of delirious patients and healthy patients after the extraction of brain parenchyma in order to qualitatively analyze the influence of delirium on the brain of patients. Affine transformation is used to align the brain, and then nonlinear registration is used to make the brain image shape of delirious patients and normal people consistent with the template. It is assumed that the image to be registered is *O* and the registered image is *C*, and the image *O* is processed by scaling, rotating, translating, and other operations through affine transformation, so that the image *O* is gradually consistent with *C*, and the process is given as follows:

(5) \[C=O\left[\begin{array}{ccc}{a}_{11} {a}_{12} {a}_{13}\\ {a}_{21} {a}_{22} {a}_{23}\\ {a}_{31} {a}_{32} {a}_{33}\end{array}\right]+\left[\begin{array}{c}{b}_{0}\\ {b}_{1}\\ {b}_{2}\end{array}\right].]\]

In affine transformation, the objective function is also used to obtain the above parameters. The objective function will be introduced later. After implementing affine transformation, since images *O* and *C* are only roughly consistent, it is necessary to continue to make images *O* and *C* more accurately coincide through nonlinear registration

(6) \[C=G(O),]\]

where *G* represents a nonlinear function. Large deformation differential homeomorphic metric mapping is mainly adopted to realize nonlinear registration, and correlation coefficient (CC) is adopted as the objective function in multimodal registration. The relationship between them is as follows:

(7) \[\text{CC}=\frac{{\displaystyle \sum }_{i=1}^{N}({C}_{i}-\bar{C})({O}_{i}-\bar{O})}{\sqrt{{\displaystyle \sum }_{i=1}^{N}{({C}_{i}-\bar{C})}^{2}}\sqrt{{\displaystyle \sum }_{i=1}^{N}{({O}_{i}-\bar{O})}^{2}}},]\]

where \[{C}_{i}]\] and \[{O}_{i}]\] mean the gray value of the *i*th pixel of the registered image and the image to be registered, \[\bar{C}]\] and \[\bar{O}]\] represent the average value of gray value, and *N* denotes the total amount of all pixels. If the CC value is at the maximum, it indicates that the two images have been accurately registered.

In addition, phase winding occurs when phase images are acquired

(8) \[\varnothing (r)={\varnothing }_{w}+2\pi J(r),]\]

where \[\varnothing (r)]\] represents the phase at space *r*, and \[(r)]\] represents the phase of winding. The obtained phase image needs to be unwrapped first. The Laplace operator is used to process phase unwrapping, that is, Fourier transform is performed on the image after the phase image is processed by the Laplace algorithm, and finally, the frequency space representation of the unwrapping phase is obtained

(9) \[\varnothing (k)=-\frac{FT({{\nabla }}^{2}\varnothing )}{4{\pi }^{2}{k}^{2}}.]\]

The Laplace operator can be expressed by Sine and Cosine symbols

(10) \[{\nabla }^{2}\varnothing =\text{cos}\varnothing {\nabla }^{2}\text{sin}\varnothing -\text{sin}\varnothing {\nabla }^{2}\text{cos}\varnothing .]\]

The relationship between the Fourier transform and Laplace in the form of a trigonometric function is given as follows:

\[{{\nabla }}^{2}\text{sin}\varnothing =-4{\pi }^{2}F{T}^{-1}{[}{k}^{2}FT(\text{sin}\varnothing )]]\]

(11) \[{{\nabla }}^{2}\text{cos}\varnothing =-4{\pi }^{2}F{T}^{-1}{[}{k}^{2}FT(\text{cos}\varnothing )].]\]

Equation (12) is obtained by combining equation (9) with equation (11):

(12) \[\varnothing (k)=KT\frac{\{\text{cos}\varnothing F{T}^{-1}{[}{k}^{2}FT(\text{sin}\varnothing )]-\text{sin}\varnothing F{T}^{-1}{[}{k}^{2}FT(\text{cos}\varnothing )]\}}{{k}^{2}},]\]

where \[{\varnothing }(k)]\] stands for the frequency space representation of the phase image after unwinding.

After phase unwrapping, there are still some interference phases caused by the background magnetic field in the phase image. The background interference is removed by variable-kernel sophisticated harmonic artifact reduction for phase data. Because the external phase of FOV has a harmonic property, its Laplace operation meets \[{{\nabla }}^{2}{\varnothing }=0]\] , so the Laplace operation can quickly remove the external phase interference of FOV. Phase interference removal in brain tissue goes through two stages. In the first stage, sphere mean value (SMV) filtering is performed to remove harmonic components

(13) \[\varnothing ^{\prime} =\varnothing -S\otimes \varnothing ,]\]

where *S* means SMV convolution kernel. The second step is to perform deconvolution to recover the local phase lost during SMV filtering

(14) \[{\varnothing }_{\text{nh}}=(\delta -S)\otimes \varnothing ^{\prime} =F{T}^{-1}\left[FT(\varnothing ^{\prime} )\cdot \left(1+\frac{FT(S)}{1-FT(S)}\right)\right].]\]

\[K=F{T}^{-1}\left[\frac{FT(S)}{1-FT(S)}\right]]\] , equation (14) is changed to equation (15)

(15) \[{\varnothing }_{\text{nh}}=\varnothing ^{\prime} +\varnothing ^{\prime} \otimes K,]\]

where \[{{\varnothing }}_{\text{nh}}]\] represents the final phase of removing the background phase.

After phase unwrapping and background phase removal, \[{{\varnothing }}_{\text{nh}}]\] carries out dipole kernel convolution inversion to obtain the magnetic susceptibility image. It is solved by least square QR-factorization (LSQR). According to the corresponding relationship between susceptibility and phase diagram, the following equation is obtained:

(16) \[\varnothing (k)\propto D(k)X(k),]\]

where \[{\varnothing }(k)]\] , \[D(k)]\] , \[\hspace{.25em}X(k)]\] are the phase diagram, dipole kernel, and the spectral space representation of magnetic susceptibility, respectively. Therefore, the solution of the magnetic susceptibility is expressed as the following optimization solution:

(17) \[\chi=\text{min}\{{F}^{-1}(D(k)X(k))-{\varnothing }_{\text{nh}}\}+\lambda R(\chi),]\]

where \[R(\chi)]\] is a regular function, the magnetic susceptibility image is constrained, and \[\lambda ]\] is the regular coefficient. \[R(\chi)]\] uses the L1 norm regularization

(18) \[R(\chi)=W{G}_{\chi}1=\sqrt{{({W}_{\chi}{G}_{\chi}\chi)}^{2}+{({W}_{\text{y}}{G}_{\text{y}}\chi)}^{2}+{({W}_{\text{z}}{G}_{\text{z}}\chi)}^{2}},]\]

\[\text{where }{W}_{\chi}]\] , \[{W}_{y}]\] , \[{W}_{z}]\] are the weight coefficients, and \[{G}_{\chi}]\] , \[{G}_{y}]\] , \[{G}_{z}]\] represent the first derivative. The magnetic susceptibility image can be obtained by iterative optimization.

### 2.4 Evaluation of reconstructed images

Root mean square error (RMSE), high-frequency error norm (HFEN), and structural similarity (SSIM) are adopted for evaluation to quantitatively evaluate the reconstructed image

(19) \[\text{RMSE}=\sqrt{\frac{{\displaystyle \sum }_{j=1}^{M}{\displaystyle \sum }_{j=1}^{N}{({x}_{ij}-{y}_{ij})}^{2}}{M\times N}}.]\]

RMSE mainly uses the pixel difference between two images to evaluate the consistency of two images. \[{x}_{ij}]\] denotes the pixel value of the *ij* coordinate of the label image, \[{y}_{ij}]\] means the pixel value of the *ij* coordinate of the network output image

(20) \[\text{HFEN}=\frac{{\displaystyle \sum }_{1}^{N}\frac{\text{log}({x}_{i})-\text{log}{({y}_{i})}_{F}^{2}}{\text{log}{({x}_{i})}_{F}^{2}}}{N},]\]

where \[\text{log}(x)]\] refers to the Laplace Gaussian filter function, *x* refers to the label image, and *y* refers to the network output image.

(21) \[\text{SSIM}=\frac{(2{\mu }_{x}{\mu }_{y}+{c}_{1})({\sigma }_{xy}+{c}_{2})}{({\mu }_{x}^{2}+{\mu }_{y}^{2}+{c}_{1})({\sigma }_{x}^{2}+{\sigma }_{y}^{2}+{c}_{2})},]\]

where \[{\mu }_{x}]\] is the mean of *x*, \[{\mu }_{y}]\] is the mean of *y*, \[{\sigma }_{x}^{2}]\] represents the variance of *x*, \[{\sigma }_{y}^{2}]\] means the variance of *y*, \[{\sigma }_{xy}]\] denotes the covariance of *x*, *y*, and \[{c}_{1}]\] , \[{c}_{2}]\] , \[{c}_{3}]\] are constants.

### 2.5 Statistical methods

All data were entered into Excel and processed using BM SPSS 22.0 software, chi-square test for qualitative data, and independent sample *t*-test for quantitative data; the differences in the obtained MRI parameters between the two groups were processed by REST software, multiple comparison correction was performed adopting Monte Carlo simulation method, and correction was performed adopting AlphaSim tool of REST software, and *P <* 0.05 was considered to indicate a statistically significant difference. REST slice viewer software can display statistically significant brain regions and extract information about each brain region.

## 3 Results

### 3.1 Comparative analysis of two groups of algorithms

**Figure 1:**
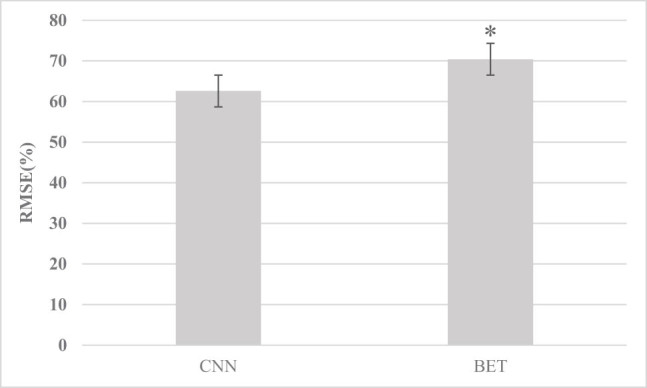
RMSE comparison between the two algorithms (* indicates comparison with the CNN algorithm, *P* < 0.05).

**Figure 2:**
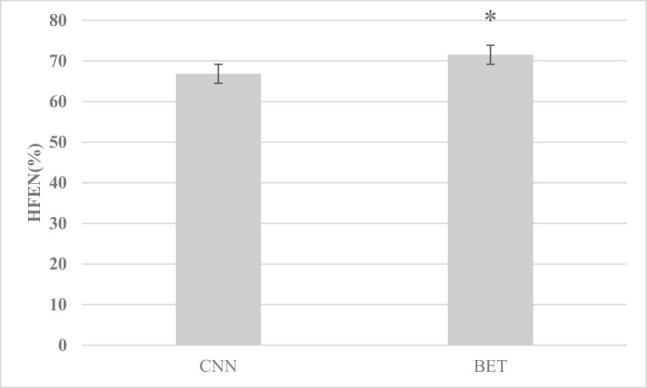
HFEN comparison between the two algorithms (* indicates comparison with the CNN algorithm, *P* < 0.05).

**Figure 3:**
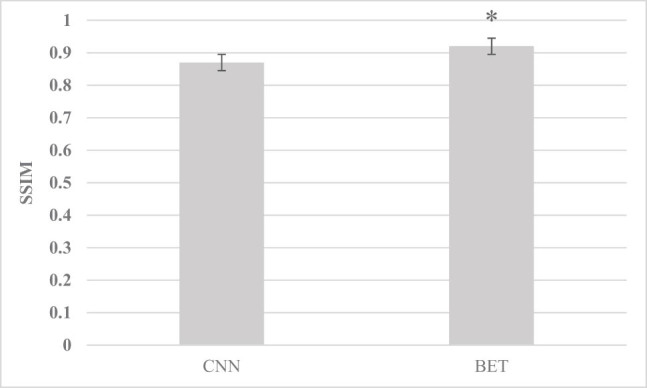
SSIM comparison between the two algorithms (* indicates comparison with the CNN algorithm, *P* < 0.05).

Figures 1–3 show that the RMSE, HFEN, and SSIM values of the images under the convolution neural network (CNN) algorithm were 62.58%, 66.83%, and 0.87, respectively, and those under the BET algorithm were 70.4%, 71.5%, and 0.92, respectively. Compared with the CNN algorithm, there was a significant difference (*P* < 0.05).

### 3.2 Comparison of brain ReHo values

ReHo values were abnormally elevated in the pontine midbrain, hippocampus (right), cerebellum (left), midbrain, and basal ganglia core in delirium patients (*P <* 0.05, AlphaSim correction); ReHo values were abnormally decreased in the superior frontal gyrus, middle frontal gyrus, and left inferior frontal gyrus, parietal lobe, and temporal lobe in delirium patients (Tables 1 and 2).

**Table 1:** Statistical results of enhanced ReHo values in delirium group

Location	*X*	*Y*	*Z*	AlphaSim correction
Pons	−8	−30	−34	*P <* 0.05
Hippocampal region (right)	−28	−40	−9	*P <* 0.05
Cerebellum (left)	−39	−60	−50	*P <* 0.05
Mesencephalon	6	−30	−14	*P <* 0.05
Basal ganglia core	−24	0	15	*P <* 0.05

Note: *X*, *Y*, *Z* are MNT coordinate axis (mm).

**Table 2:** Statistical results of decreased ReHo values in the delirium group

Location	*X*	*Y*	*Z*	AlphaSim correction
Superior frontal gyrus (left)	−25	55	10	*P <* 0.05
Superior frontal gyrus (right)	43	55	12	*P <* 0.05
Middle frontal gyrus (left)	−34	40	33	*P <* 0.05
Middle frontal gyrus (right)	35	58	21	*P <* 0.05
Inferior frontal gyrus (left)	−40	13	28	*P <* 0.05
Temporal lobe	47	21	−4	*P <* 0.05
Parietal lobe	−6	18	50	*P <* 0.05

### 3.3 Comparison of DTI detection results

Patients with delirium had evidently lower FA values in the cerebellum (left), frontal lobe, temporal lobe (left), corpus callosum, and hippocampus (left) (*P <* 0.05); patients with delirium had clearly higher MD values in the medial frontal lobe, superior temporal gyrus (right), anterior half of the cingulate gyrus, bilateral insula, and caudate nucleus (left) (*P <* 0.05) (Tables 3 and 4).

**Table 3:** Decreased FA values in the delirium group

Location	*X*	*Y*	*Z*	AlphaSim correction
Cerebellum (left)	−35	−60	−43	*P <* 0.05
Frontal lobe (left)	−28	24	17	*P <* 0.05
Frontal lobe (right)	30	25	18	*P <* 0.05
Temporal lobe (left)	−30	−20	−15	*P <* 0.05
Corpus callosum	0	9	23	*P <* 0.05
Hippocampal region (left)	−30	−25	−10	*P <* 0.05

**Table 4:** Enhanced MD values in delirium group

Location	*X*	*Y*	*Z*	AlphaSim correction
Medial frontal lobe	5	55	0	*P <* 0.05
Superior temporal gyrus (right)	45	0	−9	*P <* 0.05
Anterior half of cingulum gyrus	−1	41	20	*P <* 0.05
Insula (left)	−45	−5	−5	*P <* 0.05
Insula (right)	48	10	5	*P <* 0.05
Caudate nucleus (left)	−10	16	7	*P <* 0.05

## 4 Discussion

In recent years, the emergence of resting-state functional MRI has provided a new direction for the diagnosis and evaluation of brain diseases and continues to develop and progress in application, and DTI and MRI techniques are often commonly used for the diagnosis of diseases [16]. In addition to the introduction and improvement of deep learning algorithms and the increase of clinical needs, the research on delirium patients has also increased, and the pathogenesis of delirium has been gradually unraveled, which has a non-negligible impact in the medical field and provides an important reference for the diagnosis and treatment of delirium. In continuous clinical practice, researchers have obvious advantages in analyzing MRI in cranial nerve function, mainly in the imaging of assessing brain function structure. MRI can comprehensively and intuitively display the intracranial structure of delirium patients, the brain mechanism and metabolic mechanism of delirium patients are fully and completely displayed, and the application of deep learning algorithm also further improves the imaging quality. It lays a foundation for the application and development of imaging technology and provides a more reliable technical basis for its practical application in clinical diagnosis. Both the diagnosis of delirium and the evaluation of its subsequent treatment can play an important role. The image quality obtained by deep learning algorithm reconstruction is clearly improved.

The results showed that the RMSE, HFEN, and SSIM of the BET algorithm were 70.4%, 71.5%, and 0.92, respectively, which were significantly higher than those of the CNN algorithm (*P* < 0.05), which shows that the BET algorithm can better improve the image quality and has positive application value. Kepka et al. [17] showed that deep learning algorithms can promote the reconstruction of resting functional MRI images. Based on the BET learning model, high-speed automatic positioning and HALF technology can achieve rapid MRI reconstruction, which can provide high-resolution and high-quality resting functional MRI data. Moreover, it was found that in contrast with the normal control group, in delirium patients, the ReHo values of the pontine, hippocampus (right), cerebellum (left), midbrain, and basal ganglia core were increased in delirium patients; the ReHo values of the superior frontal gyrus, middle frontal gyrus, and left inferior frontal gyrus, parietal lobe, and temporal lobe were decreased; the FA values of the cerebellum (left), frontal lobe, temporal lobe (left), corpus callosum, and hippocampus (left) were decreased; and the MD values of the medial frontal lobe, superior temporal gyrus (right), anterior half of the cingulate gyrus, bilateral insula, and caudate nucleus (left) were increased (*P <* 0.05). The cerebellum plays a major role in controlling the coordinated movements of the extremities of the human body, and the uncoordinated movements of the extremities in delirium patients may be caused by this effect. Previous studies have also shown that changes in low-frequency fluctuation amplitude values in the right hippocampus and bilateral anterior cingulate cortex are obviously associated with changes in cognitive function in patients [18]; both the posterior cingulate cortex and the medial prefrontal cortex in delirium patients are closely connected to the dorsolateral prefrontal cortex, and the posterior cingulate cortex is hyperconnected to the inferior parietal lobule, while the medial prefrontal cortex is hyperconnected to the frontal polar cortex, hypo-connected to the superior frontal gyrus, and the connectivity between the striatum and the anterior cingulate cortex and insula is increased [19]; resting-state brain dysfunction in the left superior frontal gyrus is closely related to the occurrence of delirium, and there are outcome injuries in the frontal lobe, temporal lobe, corpus callosum, hippocampus, and cerebellum in delirium patients [20]. It is consistent with this finding.

## 5 Conclusion

This study showed that MRI based on the BET algorithm can effectively improve image quality. Patients with delirium had abnormal brain functional activity at rest in many brain regions, and abnormal brain functional activity at rest was related to delirium. However, the deficiency of this study is that the sample size is very small, and the sample size can be further increased in the future to make the research results more convincing.
